# Parental Feeding Practices in Relation to Maternal Education and Childhood Obesity

**DOI:** 10.3390/nu12041033

**Published:** 2020-04-09

**Authors:** Priscilla Ayine, Vaithinathan Selvaraju, Chandra M. K. Venkatapoorna, Thangiah Geetha

**Affiliations:** 1 Department of Nutrition, Dietetics, and Hospitality Management, Auburn University, Auburn, AL 36849, USA; pza0022@tigermail.auburn.edu (P.A.); vzs0041@auburn.edu (V.S.); iamchandru@gmail.com (C.M.K.V.); 2 Boshell Metabolic Diseases and Diabetes Program, Auburn University, Auburn, AL 36849, USA

**Keywords:** parental feeding practices, child weight status, obesity, parental perceptions

## Abstract

Parental beliefs, attitudes, and feeding practices play a vital role in childhood obesity. This study aimed to assess parental perceptions, concerns about weight, feeding practices using the Child Feeding Questionnaire (CFQ), and its association with body mass index (BMI) and maternal education in elementary school children. Participants aged 6–10 years (*n* = 169) were recruited and anthropometric measurements were obtained. Pearson’s correlation and hierarchical linear regression analysis were used to examine the association between BMI *z*-score and the seven factors of the CFQ. The BMI *z*-score was significantly associated with parental perceived child weight and concern about child weight. The BMI *z*-score had a significant negative association with parents pressuring children to eat. Parents of obese children reported significantly higher (*p* < 0.001) levels of perceived child weight (β = 0.312) and concern (β = 0.320) about their child’s weight compared to the normal weight and overweight groups. Parents of overweight children showed considerably less (β = −0.224; *p* < 0.005) pressuring towards their children to eat as compared to parents of normal weight children. Additionally, we found that the parental feeding practice (pressure to eat) was only dependent upon maternal education. The path analysis indicates that maternal education has a mediating effect on BMI *z*-score and pressure to eat is related to BMI *z*-score through maternal education. The findings demonstrate the association between the parents’ perceptions, concerns, and pressure to eat with BMI *z*-score of elementary school-aged children. Only the parental feeding practice pressure to eat was dependent upon the maternal education.

## 1. Introduction

Although childhood obesity has received significant attention over the past few decades, it remains a major public health concern [1]. In the United States, childhood obesity has tripled in the past decade. It is estimated that about 13.7 million children and adolescents are currently obese in the United States [2]. In Alabama, more than 36% of children are overweight and obese, and it is the 5th highest ranked state in obesity [3]. Obesity is typically characterized by an increase in body fat mass [4]. The common causes of childhood obesity include diet, behavioral, and genetic factors [5]. Chronic diseases such as type 2 diabetes mellitus, hypertension, and hypercholesterolemia have been established to be associated with obesity [6]. The persistence of childhood obesity into adulthood and its connection with morbidity is a major problem [7]. Additionally, childhood obesity is associated with several short and long-term health adverse effects, so it is crucial to identify early and address it with efficient methods. This includes metabolic disease [8], cardiovascular related disease [9,10,11,12], retinal and renal problems [13,14], and nonalcoholic fatty liver disease [15,16]. The transition of world nutritional changes starts with a high intake of processed food, sugary food, beverages, artificial juice, and drinks [17]. Children learn about the food context in their earlier periods from their families. Children’s first nutritional educators are their parents, and they channelize their food context and eating behavior. Parental beliefs and feeding practices play a very critical role in shaping the eating behaviors of children. The eating habits and physical activity of children are influenced mainly by parental beliefs and perceptions [18]. Furthermore, parents’ educational level has been found to affect their ability to process health information, leading to improved health-related decisions. The new health information drives their inspiration to adopt a healthy lifestyle as role models for their children [19,20]. Though maternal education level has been established to play a role in feeding practices of their children, not many studies have been conducted to examine the influence of maternal education on parents’ perceptions, concerns, and feeding practices in regard to child weight. Therefore, this study will fill the gap in information to examine the relationship between parental perception, feeding practices with children’s BMI, and maternal education using the Child Feeding Questionnaire (CFQ).

The CFQ is a self-reported instrument often used to assess parental feeding attitudes, beliefs, and susceptibility to obesity [21,22]. The association between parental feeding practice and obesity is not consistent across studies. Research conducted from the UK and Australia did not show the association between parental restriction and child weight [23,24,25]. The longitudinal study showed no correlation between pressure to eat and childhood obesity measures [25,26]. A further few studies conducted in children showed parental feeding practice varied across their ethnicity and socioeconomic status [24]. In another study, less restrictive feeding practice was positively associated with child weight status in Swedish children [27]. The systemic reviews showed the investigation of maternal feeding practice concerning children’s dietary intake, and BMI has focused on feeding practice [28,29,30]. The present study hypothesizes the existence of association between the parents’ perceptions, concerns, and feeding practices with children’s BMI *z*-score and maternal education in elementary school-aged children. The age groups (6 to 10 years) of the study participants reflect when the child matures and starts to eat outside the house environment, though they still are affected by parents’ control in their feeding practice and selection of food [31].

In the present study we asked two questions: first, to what extent are parents’ perceptions, concerns, and feeding practices related with the obesity of children? Second, are any of the associated factors correlated with maternal education? For this we investigated the association between BMI *z*-score and the seven factors of the CFQ. In addition, we examined the influence of maternal education on the factors that were significantly associated with BMI *z*-score.

## 2. Materials and Methods

### 2.1. Participants and Data Collection

Participants were 169 children aged 6-10 years from the Lee and Macon counties in Alabama. A study flyer was designed and distributed in and around the area. Participants were recruited at home schools, after-school programs, through friends, and through participant referrals. Parents interested in participating in the study reached out either by email or phone. Participants younger than 6 years and older than 10 years, with medical conditions such as diabetes, cardiovascular disease, or sleep apnea and those taking any medications were excluded from the study based on phone interview. Parents came with their children to Auburn University for anthropometric measurements and to complete questionnaires. In addition, the same participants were used to determine the relationship between obesity and sleep timing behavior [32]. Maternal education was collected and categorized as high school or less, associate’s degree, Bachelor’s degree, or graduate degree. Written consent was obtained from the parents and participants. This study was approved by the Auburn University Institutional Review Board (Protocol # 17-364 MR 1709). The appropriate sample size analyses were performed using G*Power 3.1.9.4. One way-Analysis of Variance (ANOVA) for three groups showed greater than power (1-β) = 0.80 with α = 0.05 to detect 0.25 change (effect size) in the sample groups of minimum 159 participants.

### 2.2. Anthropometric Measurements

The bodyweight of the children was measured without shoes or heavy clothing to the nearest 0.1 kg. The children’s height was measured to the nearest 0.1 cm on a calibrated scale attached to a stadiometer. BMI was defined based upon the ratio of weight to height (squared) of the participants. The children were classified according to BMI percentile charts for age and sex from the Center for Disease Control and Prevention [2] as underweight (BMI < 5th), normal weight (BMI ≥ 5th to < 85th), overweight (BMI ≥ 85th to < 95th), and obese (BMI ≥ 95th) (CDC, 2015). Based on the WHO growth reference 2007 data, BMI *z*-score was calculated using SPSS macro adjusting for age and sex [33].

### 2.3. Description of Factors in the Child Feeding Questionnaire (CFQ)

The CFQ is a tool developed to measure parents’ perceptions, concerns about child weight status, child-feeding attitudes, and practices [34]. It consists of 7 factors and 30 subscales. There are four factors to measure parents’ perception and concern about weight and three factors to determine the parents’ attitudes and feeding practices. The first factor in measuring parents’ perception and concern about weight is perceived responsibility [26], which consists of 3 subscales measuring parents’ perceived responsibility in feeding their child. The second factor is perceived parent weight (PPW), composed of 4 subscales that assess the history of the parents’ perceptions of their weight status. The third factor is perceived child weight (PCW), which measures parents’ perceptions of the weight status of their children from the time of birth. The fourth factor is the parental concern (CN), consisting of 3 subscales and assessing parental concerns about their child’s risk of being overweight. The remaining three factors measure parents’ attitudes and feeding practices. The first factor of feeding practices includes restriction (RST) which measures the extent to which parents limit their child’s access to food. The second factor is pressure to eat (PE) which assesses parents’ tendency to pressure their children to eat more food. The final factor is monitoring (MN) which measures the extent to which parents supervise their child’s eating. The abbreviations for the factors used in this manuscript are consistent with the original abbreviations presented in previous studies [27,34] with minor modifications.

### 2.4. Statistical Analysis

The data were analyzed using Statistical Package for Social Sciences (SPSS) version 25.0 for Windows, IBM, and Armonk, NY, USA. Descriptive statistics are expressed as the mean ± standard error or mean ± standard deviation (SD). *p* < 0.05 is considered as statistically significant. Analysis of variance (ANOVA) was used to compare the mean difference for participant’s age, gender, maternal education, BMI, and BMI *z*-score. The distribution of predictive factors was evaluated for multicollinearity and normality (skewness). Log-transformation was done for more skewed data of child feeding questionnaire factors and used for hierarchical regression analysis. Cronbach’s alpha was evaluated for items on each of the seven factors to assess internal consistency. Pearson’s correlation was used to determine the relationships between the mean item scores for each of the seven factors in the CFQ and BMI *z*-score.

To determine which group of factors in the CFQ would predict BMI *z*-score of children, the following variables were accounted for: gender, age, and maternal education. We ran a hierarchical linear regression with BMI *z*-score as the dependent variable. All seven factors of the CFQ were the predictors and the potential confounding variables. Based on the maternal education status, participants’ mean difference in the significant CFQ factors was analyzed by one-way ANOVA. A path analysis was conducted to identify the relation between the significant CFQ factors and BMI *z*-score. Furthermore, the direct and indirect effects were evaluated using maternal education as the mediating variable in the model. According to Kheirollahpour and Shohaimi [35], two paths are considered for testing the direct and indirect relationship in the model. One-way ANOVA was used to examine the mean difference for significant factors from the regression analysis (perceived child weight, parental concern, pressure to eat). Tukey’s test was used for post-hoc comparison between groups.

## 3. Results

The descriptive characteristics of the study population are presented in Table 1. Sixty-four and a half percent (*n* = 109) of participants were normal weight (NW), 18.9% (*n* = 32) were overweight (OW), and 16.6% (*n* = 28) were obese (OB). The ages of participants ranged from 6 to 10 years with a mean age of 8.42 years for all the participants. There were no significant differences observed in age, gender, or maternal education. However, there was a significant difference in BMI z-score of the OW and the OB group compared to NW participants. The average score for the subscales for each factor in CFQ was calculated. Cronbach alpha for the CFQ factors was calculated for internal consistency and it ranged from 0.66 to 0.94, as presented in Table 2. The minimum criteria of Cronbach alpha for acceptable reliability was above 0.60 [36].

The bivariate correlation between the CFQ factors and BMI z-score is shown in Table 3. A significant positive correlation was observed between perceived child weight (r = 0.399), parental concern (r = 0.399), and BMI z-score. The strongest correlation between factors is shown in restriction and pressure to eat (r = 0.403); monitoring (r = 0.322), parental concern and restriction (r = 0.345), monitoring (r = 0.341), perceived child weight (r = 0.218), and restriction (r = 0.238) showed a positive correlation with perceived responsibility. Perceived child weight also showed correlation with monitoring (r = 0.321), parental concern (r = 0.302), and restriction (r = 0.171). Similarly, the magnitude of coefficient in pressure to eat and monitoring (r = 0.262), perceived parent weight, and concern (r = 0.154) were reported. A significant negative correlation was observed between pressure to eat (r = −0.177) and BMI z-score.

The hierarchical linear regression model was used to examine the association between BMI z-score (dependent variable) and the seven factors of the CFQ (independent variable). Gender, age of participants, and maternal education were entered into the model in the first step to adjust the potential confounding variables, followed by entering each of the predictors separately. This was done to help us observe the predictive power of each of the predictors as presented in the R square change seen in the table. The grouping of the subscales was done by averaging the subscales of each factor according to Birch et al. 2001 and Nowicka et al. 2015 [27,34]. The overall equation was statistically significant (F_10_,_158_ = 8.665, *p* < 0.001). About 34% of the variance in the child BMI z-score was explained by the child feeding questionnaire (R^2^ = 0.341, Adj. R^2^ = 0.299). Perceived child weight (B = 7.644, β = 0.312, t = 4.275, *p* < 0.001), concern about child weight (B = 1.691, β = 0.320, t = 4.398, *p* < 0.001), and parents pressuring children to eat (B = −1.342, β = −0.224, t = −2.864, *p* = 0.005) were significant factors in predicting child weight status. The other four factors were not statistically significant, as shown in Table 4. The findings indicate that BMI z-score was positively associated with perceived child weight and parental concern and negatively associated with pressure to eat.

**Figure 1:**
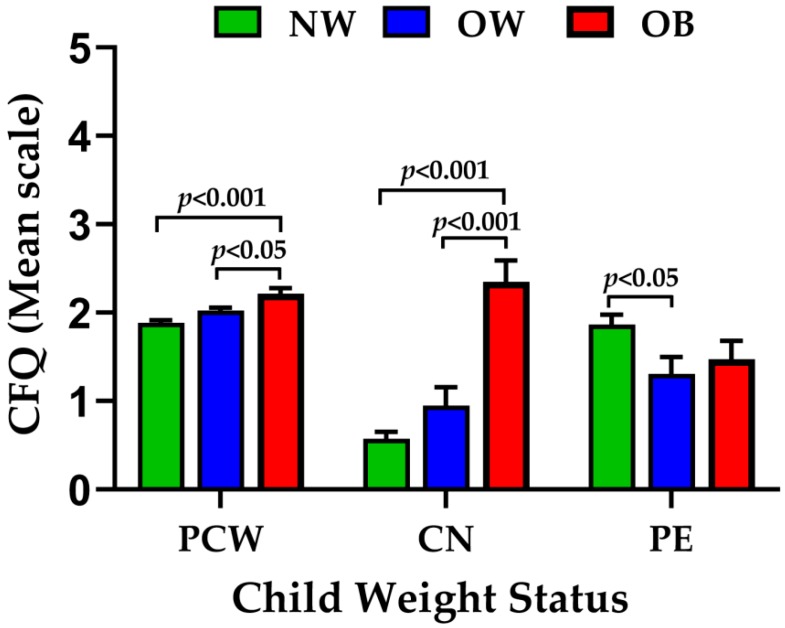
Bar graph showing the relation between the perceived child weight (PCW), parental concern (CN), and pressure to eat (PE) factors with the NW, OW, and OB participants. The values in the graph represent mean ± SEM.

Since perceived child weight, parental concern, and pressure to eat were significantly associated with BMI z-score, we further compared the means of these significant predictors with the NW, OW, and OB groups using one-way ANOVA. Parents of children in the OB category reported significantly higher (*p* < 0.0001) levels of perceived child weight and parental concern compared to the NW and OW group. Parents of OW children showed considerably less (*p* < 0.05) pressuring their children to eat as compared to NW (Figure 1).

The relationship of these significant factors, perceived child weight, parental concern, and pressure to eat, with maternal education was determined. The one-way ANOVA result (Table 5) indicated that the children of mothers with high school or less education showed significantly high pressure to eat compared to other maternal education categories.

**Figure 2:**
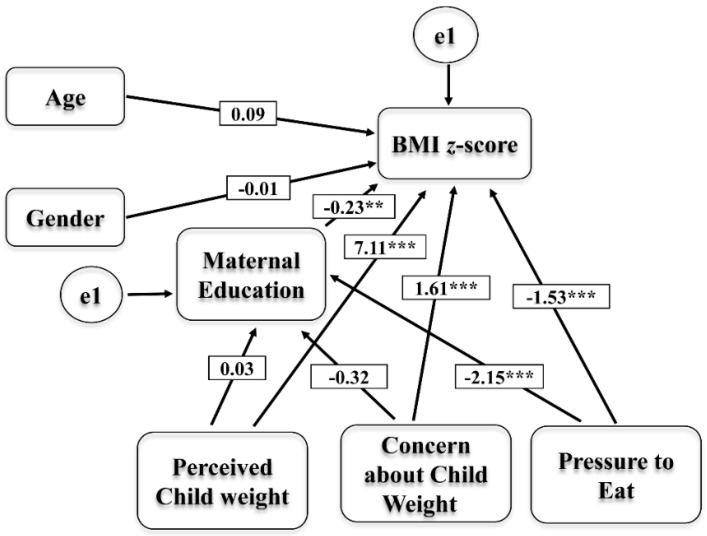
Path model showing the direct and indirect relation of the Perceived Child Weight, Parental Concern, and Pressure to Eat factors with BMI z-score through maternal education. Significant levels are ** *p* < 0.01 and *** *p* < 0.0001.

These significant factors were further confirmed by the path model which provided an acceptable fit to the data (χ^2^ = 31.786, df = 12, *p* = 0.001, CFI = 0.812, RMSEA = 0.09) [37]. As presented in Figure 2, pressure to eat had a significant negative relation with maternal education (B = −1.53, *p* < 0.001) and BMI z-score (B = −2.15, *p* < 0.001). Maternal education had a negative relation with child BMI z-score (B = -0.23, *p* = 0.002). Parental concern (B = 1.61, *p* < 0.001) and perceived child weight (B = 7.11, *p* < 0.001) had a positive relation with child BMI z-score and no significant relation with maternal education. In the model, the largest effect was observed from PE to maternal education. To test the direct and indirect effects, the three significant factors (perceived child weight, parental concern, and pressure to eat) were tested from the regression model through maternal education as a mediating variable. Table 6 shows that pressure to eat solely indirectly predicted BMI z-score through maternal education (B = 0.49, *p* < 0.002) and maternal education directly predicted BMI z-score (B = −0.23).

## 4. Discussion

This study uses the CFQ as a tool to assess the relationship between the parent’s perception, concerns, and feeding practices with the children’s BMI *z*-score and maternal education. The internal reliability determined by Cronbach’s alpha was acceptable to excellent for the factors used. Three out of the seven factors (PCW, CN, PE) of the CFQ were found to be significant predictors of child BMI *z*-score using Pearson’s correlation analysis. The perceived child weight and parental concern measure the parental perceptions and concerns about child weight. The perceived child weight and parental concern were positive indicators of child BMI *z*-score. Pressure to eat, measuring the parental feeding practices, was negatively influenced by the BMI *z*-scores, as reported by several other studies [38,39,40,41]. We found that more parents perceive their child’s weight and show a significantly higher level of concern when their child is obese, which is similar to other studies [42,43,44]. Furthermore, parents place less pressure on their overweight and obese children to eat compared to normal weight children [24,27,38]. The parental beliefs, attitudes, and feeding practices can be altered to reduce obesity [41]. However, in several studies, there are discrepancies in the relationship between parental feeding practices and obesity. Other studies found restriction to be a significant predictor of child BMI *z*-score. Restriction was not a significant predictor in our study and this may be due to the age difference of the participants [27,38]. However, no relationship is found between parental restriction and obesity in studies conducted in Australia and the United Kingdom [23,25,45]. Similarly, the pressure to eat is found not to be associated with obesity in longitudinal studies [25,26]. In agreement with our results, the correlation analysis found that BMI z-score was negatively correlated with pressure to eat among children aged 2–4 years [46]. Populations from low-income are known to be affected more by obesity [47,48]. Education may offer mothers with the awareness of the importance of nutrition in health, knowledge of children’s weight status as a risk factor for health problems in future, and maintains a good feeding practice to help in keeping a healthy weight [49]. The relationship between socio-economic status and parental feeding practices has been studied, but the results are not consistent [25,50,51]. Hence, the impact of maternal education was evaluated on the factors that were significantly associated with obesity: perceived child weight, parental concern, and pressure to eat. The parental feeding practice pressure to eat was only dependent upon maternal education. Mothers with a high school or less education pressurized their children to eat more when compared to mothers with higher education levels. This was confirmed with the path model showing that perceived child weight, parental concern, and pressure to eat were significantly associated with BMI *z*-score, but PE was only significantly related to maternal education. The study conducted by Nowicka et al 2014, describes that child BMI had direct relation to restriction, whereas pressure to eat was influenced by parental education [27]. The path model indicated that maternal education had a mediating and a direct effect on BMI z-score. Pressure to eat is related to BMI z-score and to a certain extent through maternal education. Maternal education may play a vital important role in the parental feeding practices and BMI of children. A study carried out by Cardel et al. (2012) found that socioeconomic status was negatively associated with restriction and pressure to eat [24], but our results showed maternal education was inversely related only with pressure to eat. The findings of this current study should be interpreted under this limitation. Although participants were recruited from Lee and Macon counties in Alabama, the sample cannot be considered a representation of Alabama children. The results are based upon a relatively small number of participants and a low percentage of obese participants. Another limitation of this study is that it is a cross-sectional study, but not a longitudinal study. The information obtained from these studies may not be sufficient to understand the disease trend. The cross-sectional study design provides us information about the prevalence of disease outcomes to design the cohort or longitudinal studies and gives the estimates to study the association of this study.

## 5. Conclusions

This study has furthered scientific understanding of the relation between parental perceptions, concerns, and feeding practices with the child’s obesity and maternal education. Our findings show that perceived child weight and parental concern were positively associated with BMI z-score. And the pressure to eat was negatively associated with BMI z-score of elementary school-aged children. Out of these factors, the parental feeding practice pressure to eat was only dependent upon maternal education.

## Figures and Tables

**Table 1:** Descriptive characteristics of study population.

	NW	OW	OB	*p* Value
All	109 (64.5%)	32 (18.9%)	28 (16.6%)	
Age (y)	8.37 ± 1.44	8.28 ± 1.29	8.77 ± 1.46	0.349
Gender (%)				
Male	50.46%	50.0%	42.86%	
Female	49.54%	50.0%	57.14%	
Maternal Education (%)				
High School or less	23.85%	15.63%	32.14%	
Associate Degree	23.85%	21.88%	32.14%	
Bachelor’s Degree	20.18%	28.13%	21.43%	
Graduate	32.11%	34.38%	14.29%	
BMI *z*-score	−0.02 ± 0.82	1.56 ± 0.26	2.56 ± 0.37	*p* < 0.001

NW = Normal Weight; OW = Overweight and OB = Obese; BMI = Body mass index. The values are represented as mean ± SD. Part of the data presented in the table was used in our previously published article [32].

**Table 2:** Cronbach Alpha of factors of the CFQ.

Factor	Mean ± SD	Cronbach Alpha
PR	3.41 ± 0.70	0.89
PPW	2.23 ± 0.41	0.66
PCW	1.97 ± 0.31	0.75
CN	0.94 ± 1.19	0.87
RST	2.59 ± 0.97	0.83
PE	1.69 ± 1.18	0.80
MN	2.89 ± 1.12	0.94

Perceived Responsibility (PR), Perceived Parent Weight (PPW), Perceived Child Weight (PCW), Parental Concern about Child Weight (CN), Restriction (RST), Pressure to Eat (PE), and Monitoring (MN).

**Table 3:** Pearson’s correlations between CFQ factors and BMI *z*-score.

	BMI *z*-Score	PR	PPW	PCW	CN	RST	PE	MN
BMI *z*-score	1.000							
PR	−0.031	1.000						
PPW	0.122	0.008	1.000					
PCW	0.399 **	0.218 **	0.062	1.000				
CN	0.399 **	0.115	0.154 *	0.302 **	1.000			
RST	0.052	0.238 **	0.068	0.171 *	0.345 **	1.000		
PE	−0.177 *	0.129	−0.073	−0.065	0.080	0.403 **	1.000	
MN	0.038	0.341 **	−0.062	0.321 **	0.116	0.322 **	0.262 **	1.000

Body mass index (BMI), Perceived Responsibility (PR), Perceived Parent Weight (PPW), Perceived Child Weight (PCW), Parental Concern about Child Weight (CN), Restriction (RST), Pressure to Eat (PE), and Monitoring (MN). * Correlation is significant at the 0.05 level (2-tailed). ** Correlation is significant at the 0.01 level (2-tailed).

**Table 4:** Hierarchical linear regression analyses for BMI z-scores on CFQ factors.

CFQ Factor	B-Value	Change in R^2^	β (95% CI min, max)	*p* Value
PR	−1.562	0.001	−0.108 (−1.669, 1.453)	0.130
PPW	1.105	0.016	0.050 (1.155, −1.055)	0.454
PCW	7.644	0.165	0.312 (7.956, −7.332)	**< 0.001**
CN	1.691	0.069	0.320 (2.009, −1.369)	**< 0.001**
RST	−0.531	0.014	−0.063 (−0.595, 0.469)	0.427
PE	−1.342	0.034	−0.224 (−1.564, 1.116)	**0.005**
MN	0.164	0.000	0.023 (0.187, −0.141)	0.767

The results were adjusted for the child’s gender, age, and maternal education. Perceived Responsibility (PR), Perceived Parent Weight (PPW), Perceived Child Weight (PCW), Parental Concern about Child Weight (CN), Restriction (RST), Pressure to Eat (PE), Monitoring (MN). Statistically significant factors were represented in bold.

**Table 5:** One-way ANOVA of CFQ factors score with respect to the maternal education and weight categories.

Maternal Education	PCW	CN	PE
High School or less	1.94 (0.19)	0.87 (1.03)	2.56 (1.18)
Associate Degree	1.94 (0.37)	1.35 (1.38)	1.88 (1.18) *
Bachelor’s Degree	2.09 (0.36)	0.74 (1.10)	1.09 (0.96) **
Graduate	1.92 (0.30)	0.79 (1.16)	1.29 (0.88) **

Data are expressed as mean (SD); * *p* < 0.05 and ** *p* < 0.0001 are considered significant compared to the High School or less group. Perceived Child Weight (PCW), Parental Concern about Child Weight (CN), Pressure to Eat (PE).

**Table 6:** Perceived Child Weight, Concern about Child Weight, and Pressure to Eat Related to BMI z-score through Maternal Education.

Parameters	Model	Variables	B	*p*-Value
Perceived Child Weight	Direct Relationship	Perceived Child Weight → BMI *z*-score	7.11	0.008
	Indirect Relationship	PCW → Maternal Education → BMI *z*-score	−0.007	0.992
	Total effect	Direct + Indirect	7.10	0.008
Concern about Child Weight	Direct Relationship	CN → BMI z-score	1.61	0.004
	Indirect Relationship	CN → Maternal Education → BMI z-score	0.074	0.262
	Total effect	Direct + Indirect	1.69	0.004
Pressure to Eat	Direct Relationship	PE → BMI z-score	−1.53	0.004
	Indirect Relationship	PE → Maternal Education → BMI z-score	0.49	0.002
	Total effect	Direct _+_ Indirect	−1.04	0.016

B = Unstandardized parameter estimates. Perceived Child Weight (PCW), Parental Concern about Child Weight (CN), Pressure to Eat (PE).
